# Bioactivity of Glass Carbomer Versus Conventional GICs in Sound Enamel and Dentine: A 12-Month SEM-EDS Study

**DOI:** 10.3390/ma18153580

**Published:** 2025-07-30

**Authors:** Dubravka Turjanski, Suzana Jakovljević, Dragutin Lisjak, Petra Bučević Sojčić, Fran Glavina, Kristina Goršeta, Domagoj Glavina

**Affiliations:** 1 Department of Paediatric and Preventive Dentistry, Dental Polyclinic Zagreb, Perkovčeva 3, 10000 Zagreb, Croatia; 2 Faculty of Mechanical Engineering and Naval Architecture, University of Zagreb, Ivana Lučića 5, 10000 Zagreb, Croatia; suzana.jakovljevic@fsb.unizg.hr (S.J.); dragutin.lisjak@fsb.unizg.hr (D.L.); 3 Department of Paediatric and Preventive Dentistry, School of Dental Medicine, University of Zagreb, Gundulićeva 5, 10000 Zagreb, Croatia; pbucevic@sfzg.unizg.hr (P.B.S.); gorseta@sfzg.unizg.hr (K.G.); glavina@sfzg.unizg.hr (D.G.); 4 ART Dental—Dental Clinic, Tometici 1d, 51215 Kastav, Croatia; fran.glavina98@gmail.com

**Keywords:** glass carbomer, glass ionomer cement, ion release, hydroxyapatite nanoparticles, SEM, EDS, fluoride uptake, mineralisation, sound dentine, sound enamel

## Abstract

Glass ionomer cements (GICs) are bioactive restorative materials valued for their sustained ion release and remineralisation capacity. However, their long-term interactions with sound enamel and dentine remain underexplored. This 12-month in vitro study aimed to evaluate microstructural and compositional changes in sound dental tissues adjacent to four GICs—Ketac Universal, Fuji IX and Equia Forte Fil (conventional GICs) and the advanced Glass Carbomer (incorporating hydroxyapatite nanoparticles)—using field-emission scanning electron microscopy (FE-SEM) and energy-dispersive X-ray spectroscopy (EDS). Glass Carbomer uniquely formed hydroxyapatite nanoparticles and mineralised regions indicative of active biomineralisation—features not observed with conventional GICs. It also demonstrated greater fluoride uptake into dentine and higher silicon incorporation in both enamel and dentine. Conventional GICs exhibited filler particle dissolution and mineral deposition within the matrix over time; among them, Equia Forte released the most fluoride while Fuji IX released the most strontium. Notably, ion uptake was consistently higher in dentine than in enamel for all materials. These findings indicate that Glass Carbomer possesses superior bioactivity and mineralising potential which may contribute to the reinforcement of sound dental tissues and the prevention of demineralisation. However, further in vivo studies are required to confirm these effects under physiological conditions.

## 1. Introduction

Glass ionomer cements (GICs) are widely used in restorative dentistry due to their unique properties including chemical adhesion to dental hard tissues, sustained fluoride release, biocompatibility and ease of clinical handling [1,2]. These characteristics make GICs suitable for various clinical applications such as restorative fillings, liners, luting agents, sealants and atraumatic restorative treatment (ART), particularly in paediatric, geriatric and community dental care settings [3,4]. Bioactive glasses such as Bioglass^®^ were initially developed for their ability to chemically bond with biological tissues, a feature that laid the foundation for GICs with similar chemical properties [1]. GICs release ions that facilitate remineralisation and chemical bonding, thereby playing an indispensable role in restorative dentistry. Recognised for their bioactivity, GICs form stable chemical bonds with enamel and dentine, release biologically active ions and promote mineral deposition through local pH modulation at the material–tissue interface [5,6].

However, traditional GICs exhibit limitations in mechanical durability, aesthetic performance and wear resistance, restricting their use primarily to non-stress-bearing areas or temporary restorations [7]. To address these drawbacks, modifications involving nanoparticles, bioactive glass fillers and hydroxyapatite (HA) particles have been introduced. Modern GIC formulations release multiple therapeutic ions including fluoride, calcium, aluminium, silicon, phosphorus and strontium, each providing distinct biological benefits [8,9,10,11]. Fluoride ions facilitate enamel remineralisation through the formation of fluorohydroxyapatite, increasing enamel resistance to acid-induced demineralisation [9]. Strontium ions further enhance enamel stability by substituting calcium in the hydroxyapatite lattice, thereby reducing enamel solubility and improving resistance to cariogenic challenges [12].

Recently, advanced GICs such as Glass Carbomer, enriched with hydroxyapatite nanoparticles, have emerged offering improved mechanical strength, enhanced bioactivity and greater therapeutic ion release compared to conventional formulations [13,14]. However, most existing studies have focused predominantly on short-term effects or interactions with demineralised tooth structures, while long-term interactions with sound non-demineralised enamel and dentine—defined here as healthy, intact dental hard tissues without signs of demineralisation or carious lesions—remain less well understood [15,16,17]. Comprehensive data on elemental exchange, bioactive potential and microstructural changes of advanced GIC formulations in contact with intact dental tissues remain limited.

Investigating these long-term interactions is essential as the incorporation of bioactive ions into sound dental tissues may significantly improve their resistance to future acid attacks, thus preventing initial demineralisation and potentially reducing the risk of secondary caries [13,18,19,20].

The present study aims to investigate the microstructural and compositional evolution of GICs and their interface with adjacent non-demineralised dental tissues over a 12-month period. Using field-emission scanning electron microscopy (FE-SEM) and energy-dispersive X-ray spectroscopy (EDS), the objectives are to:

- Characterise microstructural changes in GICs over 12 months;
- Monitor elemental composition changes in GICs during this period;
- Assess compositional modifications in non-demineralised enamel adjacent to GICs;
- Evaluate chemical changes in non-demineralised dentine near the materials.

This research hypothesises that Glass Carbomer, due to its advanced composition, will exhibit superior bioactivity including enhanced ion uptake and the formation of mineralised regions compared to conventional GICs. The outcomes of this study could inform restorative strategies aimed at strengthening healthy dental tissues and advancing preventive dentistry.

## 2. Materials and Methods

### 2.1. Study Design

This in vitro study was approved by the Ethics Committee of the School of Dental Medicine, University of Zagreb (approval number: 05-PA-24-2/2018). Intact human molars were obtained from the Department of Oral Surgery at the same institution. The teeth were extracted for clinical reasons unrelated to this study; therefore, informed consent was not required. A total of 12 molars were used, divided into 4 groups corresponding to the tested glass ionomer materials. Four encapsulated glass ionomer materials were investigated using field-emission scanning electron microscopy (FE-SEM) combined with energy-dispersive X-ray spectroscopy (EDS):

- Ketac Universal Aplicap (3M ESPE, Seefeld, Germany), a conventional glass ionomer cement composed primarily of fluoroaluminosilicate glass powder and a liquid phase containing polyalkenoic acid copolymers and tartaric acid [21];
- Fuji IX GP Fast (GC Corporation, Tokyo, Japan), a high-viscosity conventional GIC containing strontium fluoroaluminosilicate glass and polyacrylic acid [22];
- Equia Forte Fil (GC Corporation, Tokyo, Japan), a resin-coated high-viscosity conventional GIC combining strontium fluoroaluminosilicate glass with a resin coating for improved wear resistance [23];
- Glass Carbomer GlassFill (GCP Dental, Vianen, The Netherlands), an enhanced formulation incorporating fluoroaluminosilicate glass, hydroxyapatite nanoparticles and bioactive glass [13].

### 2.2. Sample Preparation

Sample preparation was performed on the extracted molars. Crowns were separated from roots and standardised round cervical cavities (2 mm diameter × 2 mm depth) were prepared on both buccal and lingual surfaces using an inverse-conical diamond bur under water cooling. The cavities were conditioned with 5% sodium hypochlorite (NaOCl; Gram-Mol d.o.o., Zagreb, Croatia) for 5 s, rinsed with water and air-dried. Materials were applied according to manufacturers’ instructions, isolated with petroleum jelly and thermo-cured for 60 s using a polymerisation unit without active cooling (Bluephase G2, Ivoclar Vivadent, Schaan, Liechtenstein; 1200 mW/cm^2^).

Following setting, specimens of each material were stored separately in artificial saliva for durations of 1 week, 6 months and 12 months to prevent cross-contamination and ensure independent ion exchange profiles. The artificial saliva contained 0.5 g/L NaCl, 4.2 g/L NaHCO_3_, 0.03 g/L NaNO_3_ and 0.2 g/L KCl. The solution was refreshed weekly to maintain ionic stability and minimise microbial growth. After storage, the crowns were embedded in standard cylindrical moulds using epoxy resin (Clarocit, Struers, Ballerup, Denmark) and sectioned to a thickness of 2 mm using a low-speed precision cutter (Minitom, Struers, Ballerup, Denmark; 150 rpm) under water cooling.

### 2.3. Sample Size and Statistical Power

A total of 24 molars were prepared, with 12 used for analysis (n = 3 per group) and an additional 12 prepared as reserves—3 per group—to account for potential loss or damage during sectioning and handling. The analytical sample size (n = 3 per group) was selected to ensure adequate statistical power (1 − β ≥ 0.80) to detect medium to large differences in chemical concentrations among the four material groups. The effect size was estimated using Cohen’s f. This sample size was deemed sufficient to identify statistically significant differences at α = 0.05. Power analysis confirmed the study’s capacity to minimise Type II errors.

### 2.4. FE-SEM/EDS Methodology

**Figure 1:**
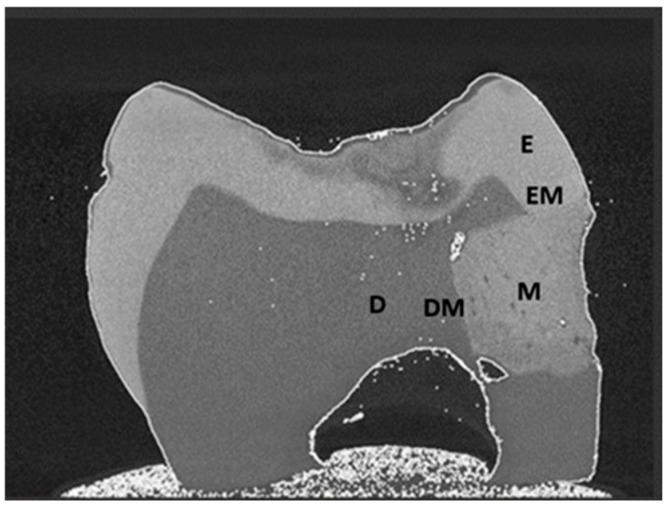
Computed tomography (CT) scan of a sample showing the locations where EDS analyses were conducted: E—enamel distant from the material; D—dentine distant from the material; M—restorative material; EM—enamel adjacent to the material; DM—dentine adjacent to the material.

The FE-SEM/EDS methodology employed in this study closely follows that described in previous research analysing the elemental composition and microstructure of dental materials [24,25,26]. SEM and EDS analyses were carried out using a field emission scanning electron microscope (FE-SEM; JSM-7000F, JEOL, Tokyo, Japan) equipped with an INCA Energy 350 energy-dispersive X-ray spectrometer (EDS; Oxford Instruments, Belfast, UK). All samples were sputter-coated with a thin layer of gold–palladium (90% Au, 10% Pd) to enhance conductivity during SEM imaging. The coating was applied using a Gatan 682 Precision Etching and Coating System (PECS; Gatan Inc., Pleasanton, CA, USA). The coating thickness was set to 5 nm and monitored using a quartz crystal microbalance integrated within the PECS. A computed tomography (CT) scan of a representative sample was used to illustrate the locations selected for EDS analysis (Figure 1).

For FE-SEM analysis, imaging was conducted at an accelerating voltage of 10 kV. Overview microstructural analysis of enamel, dentine and restorative material was performed at 300× magnification, while interface analyses between enamel/material and dentine/material were conducted at 2000× magnification. Image scale bars were set at 10 µm.

Elemental quantification was performed using standard calibration curves for each element. Chemical compositions were calculated using INCA Energy Dispersive X-ray Spectroscopy software, version 2.1 (Oxford Instruments, Belfast, UK), which applies background correction and reports elemental concentrations as weight percentages (wt.%). The elements monitored during analysis included fluoride (F), aluminium (Al), silicon (Si), phosphorus (P), calcium (Ca) and strontium (Sr).

For EDS analysis, a single measurement of a larger field (300 × 250 µm at 300× magnification) was performed for each sample area:

- E (enamel distant from the material);
- D (dentine distant from the material);
- M (restorative material).

In addition, three repeated measurements of smaller fields (45 × 15 µm, extending 45 µm along the interface and 15 µm into enamel or dentine, at 2000× magnification) were conducted for each interface region:

- EM (enamel adjacent to the material);
- DM (dentine adjacent to the material).

All EDS results were expressed in weight percentages (wt.%) [24,25,26].

### 2.5. Statistical Analysis

To evaluate temporal and spatial differences in elemental composition (F, Al, Si, P, Ca, Sr), both parametric and non-parametric statistical methods were employed. Descriptive statistics, including means, standard deviations and 95% confidence intervals, were calculated.

One-way analysis of variance (ANOVA) was used to test for statistically significant differences between material groups. The assumptions of normality and homogeneity of variances were assessed using the Shapiro–Wilk and Levene’s tests, respectively. Effect size was estimated with Cohen’s f, and statistical power (1 − β) was calculated to evaluate the reliability of the observed differences. Post hoc pairwise comparisons were conducted using Tukey’s HSD test.

When the assumptions for ANOVA were not met (e.g., non-normal distribution, unequal variances or small sample size), non-parametric alternatives, including the Kruskal–Wallis test and bootstrap resampling, were applied. Statistical significance was set at *p* < 0.05.

All statistical analyses, including both primary and post hoc comparisons, as well as graphical and numerical data processing, were performed using the MathWorks^®^ Statistics and Machine Learning Toolbox (R2023b).

## 3. Results

### 3.1. FE-SEM Analysis of Material Microstructure

**Figure 2:**
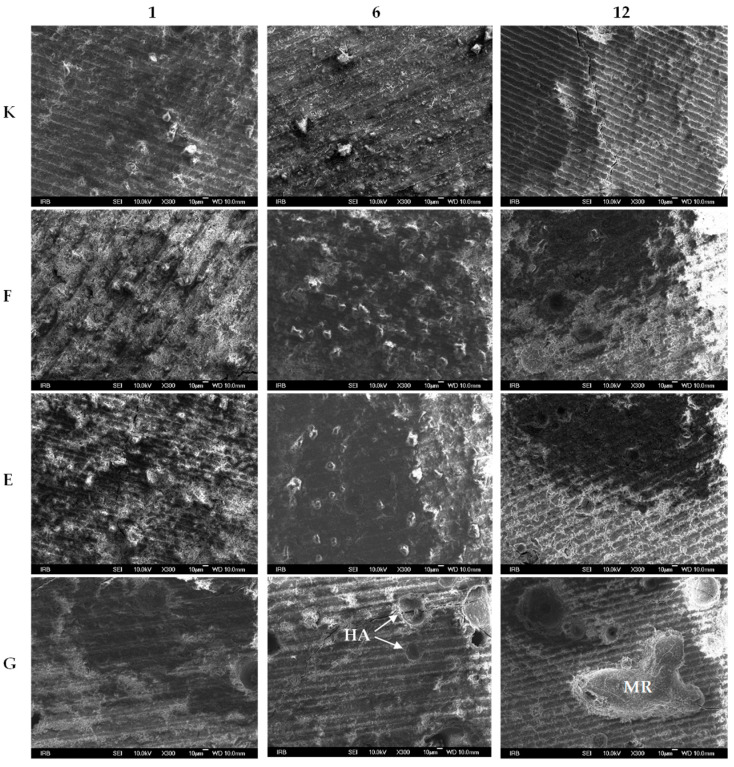
Representative FE-SEM micrographs of Ketac Universal (K), Fuji IX (F), Equia Forte (E) and Glass Carbomer (G) after 1 week (1), 6 months (6) and 12 months (12) of maturation.

Figure 2 shows representative FE-SEM micrographs of Ketac Universal (K), Fuji IX (F), Equia Forte (E) and Glass Carbomer (G) after 1 week (1), 6 months (6) and 12 months (12) of maturation.

At one week (K1, F1, E1), numerous filler particles of varying morphologies and sizes, predominantly angular and irregularly shaped, are clearly observed embedded within the organic cement matrices. By six months (K6, F6, E6), a noticeable reduction in particle density is evident. At twelve months (K12, F12, E12), the number of visible particles markedly decreases, accompanied by pronounced homogenisation of the matrix. Notably, images F12 and E12 reveal the development of a distinct mesh-like mineral network.

In Glass Carbomer at six months (G6), hydroxyapatite (HA) particles are distinctly identifiable by their rounded morphology, uniform size and smooth crystalline surfaces. At twelve months (G12), a discrete localised mineralised region (MR) appears, characterised by the cohesive fusion of multiple HA particles and radial plate-like mineral growth extending into the surrounding matrix.

Minor cracks observed throughout the samples are attributed to mechanical stresses incurred during sample preparation, including cutting and drying.

### 3.2. EDS Analysis of Chemical Composition Changes Within the Materials

**Figure 3:**
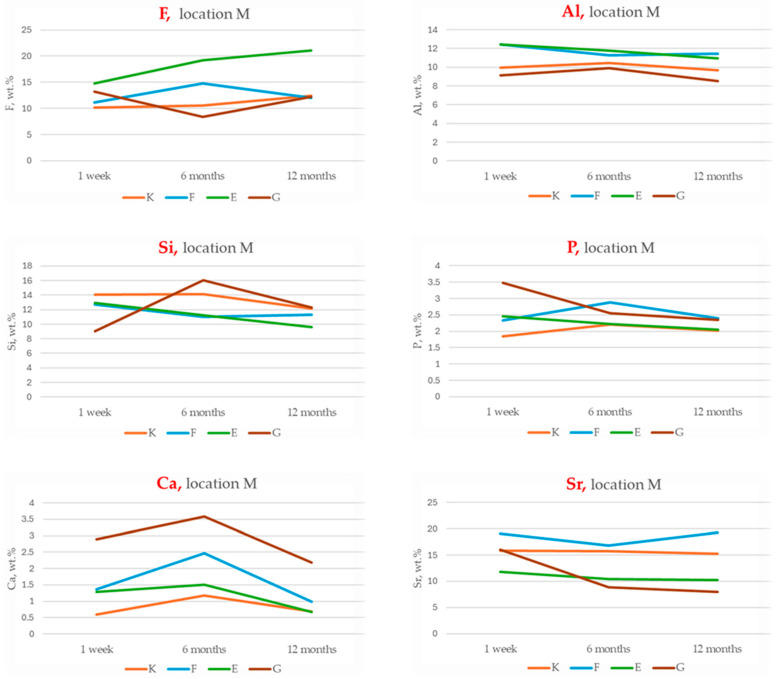
Temporal changes in chemical compositions (wt.%) within the material region (location M) of Ketac Universal (K), Fuji IX (F), Equia Forte (E) and Glass Carbomer (G) after 1 week (1), 6 months (6) and 12 months (12) of maturation.

**Figure 4:**
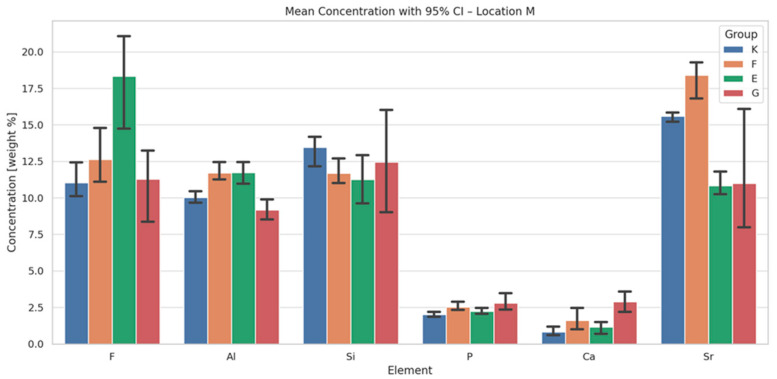
Mean chemical compositions (wt.%) of F, Al, Si, P, Ca and Sr in glass ionomer cement (location M) averaged across all time points during the 12-month observation period, with 95% confidence intervals (CIs) and standard deviations (SDs).

**Figure 5:**
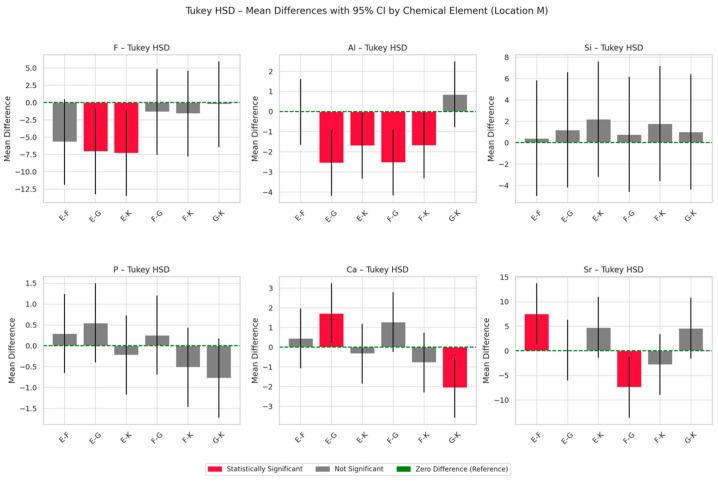
Tukey HSD mean differences with 95% confidence intervals (CIs) by chemical element in the material region (location M), averaged across all time points during the 12-month observation period.

The results of energy-dispersive spectroscopy (EDS) performed on the material region (designated as location M) of the four tested glass ionomer cements are presented in Figure 3, Figure 4 and Figure 5. Additional data are available in the Supplementary Materials (Tables S1 and S2).

Fluoride (F): Equia Forte (E) exhibits the highest initial fluoride content and shows a statistically significant increase over time (*p* < 0.05; Tukey HSD: E–K and E–G differences significant). Fuji IX (F) also demonstrates an increase in fluoride content, whereas Glass Carbomer (G) shows a marked decrease at 6 months, suggesting an initial burst release followed by depletion.

Aluminium (Al): Initial aluminium levels are significantly higher in E and F, with all materials showing a time-dependent decline (*p* < 0.001). Tukey’s post hoc test confirms significant group differences (E–K, F–G).

Silicon (Si): Although ANOVA does not detect statistically significant intergroup differences, G displays a substantial increase in silicon content after 6 months (+77.6%).

Calcium (Ca) and phosphorus (P): Calcium exhibits a biphasic trend across all materials—an initial increase followed by a subsequent decline—most pronounced in G and E. Statistically significant differences are observed between G and K (Tukey HSD, *p* < 0.05). Phosphorus levels show minimal variation; K and F maintain stable or slightly increasing concentrations, while E and G demonstrate a gradual decrease.

Strontium (Sr): Fuji IX (F) consistently exhibits the highest strontium levels at all time points, with statistically significant differences compared to G and E (*p* < 0.05).

### 3.3. EDS Analysis of Elemental Composition Changes in the Enamel Adjacent to the Material

Descriptive and inferential statistical methods were applied to examine temporal trends and inter-material differences in the concentrations (wt.%) of key ions—fluoride (F), aluminium (Al), silicon (Si), phosphorus (P), calcium (Ca) and strontium (Sr)—in enamel adjacent to four glass ionomer cements: Ketac Universal (K), Fuji IX (F), Equia Forte (E) and Glass Carbomer (G). These findings provide insight into ion exchange processes at the tooth–material interface, reflecting the biointeractivity and potential remineralisation capacity of each GIC formulation over the 12-month observation period.

**Figure 6:**
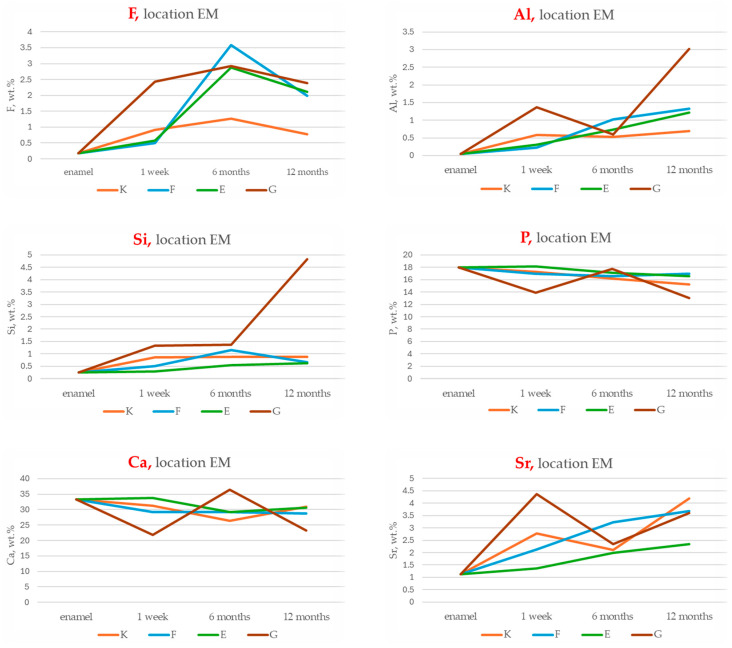
Temporal changes in chemical compositions (wt.%) of fluoride (F), aluminium (Al), silicon (Si), phosphorus (P), calcium (Ca) and strontium (Sr) in enamel adjacent to restorative materials (location EM) for Ketac Universal (K), Fuji IX (F), Equia Forte (E) and Glass Carbomer (G) after 1 week, 6 months and 12 months. Initial enamel composition (enamel) is shown as the baseline reference.

**Figure 7:**
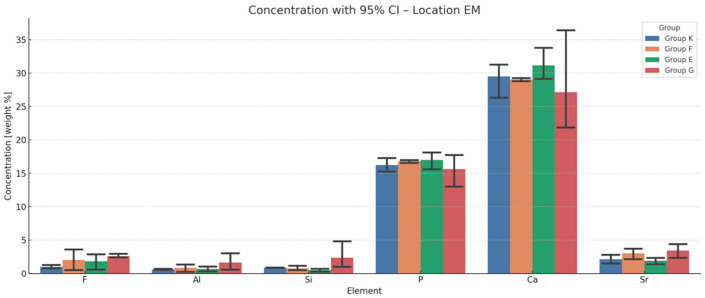
Mean chemical compositions (wt.%) of F, Al, Si, P, Ca and Sr in glass ionomer cement (location EM) averaged across all time points during the 12-month observation period with 95% confidence intervals (CIs) and standard deviations (SDs).

**Figure 8:**
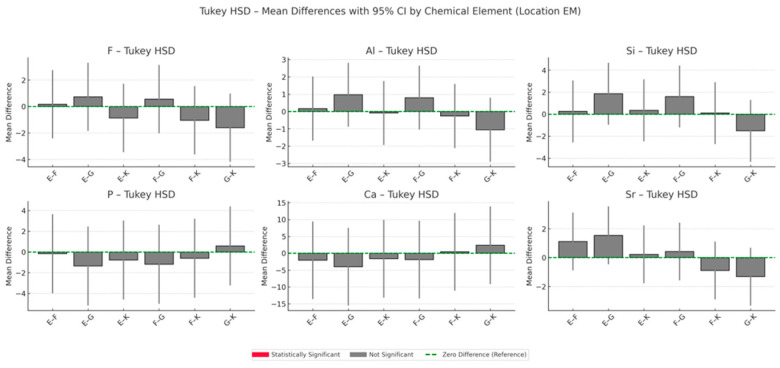
Tukey HSD mean differences with 95% confidence intervals (CIs) by chemical element in the enamel region adjacent to the restoration (location EM), averaged across all time points during the 12-month observation period.

**Figure 9:**
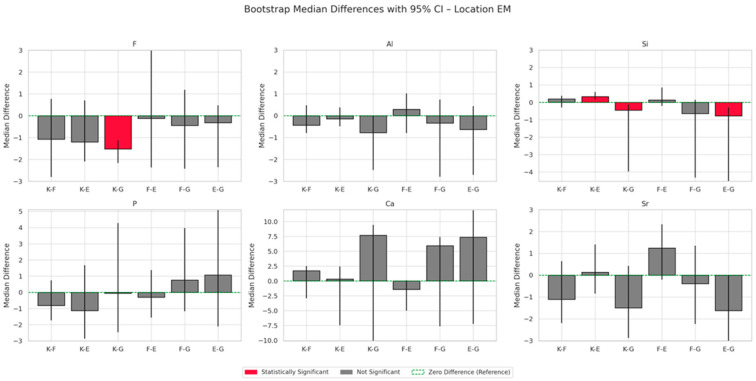
Bootstrap analysis median differences with 95% confidence intervals (CIs) by chemical element in the enamel region adjacent to the restoration (location EM), averaged across all time points during the 12-month observation period.

The results of energy-dispersive spectroscopy (EDS) performed on enamel adjacent to the restorative materials (location EM) are summarised in Figure 6, Figure 7, Figure 8 and Figure 9. Additional data are available in the Supplementary Materials (Tables S3 and S4).

Fluoride (F): At all time points, fluoride concentrations in enamel adjacent to the materials are markedly higher than those in native enamel. The greatest increase occurs at six months, with Fuji IX (F) showing the highest relative elevation (+1888.9%), followed by Glass Carbomer (+1527.8%), Equia Forte (+1494.4%) and Ketac Universal (+605.6%). Although fluoride levels decline slightly after six months, concentrations at twelve months remain elevated compared to baseline. One-way ANOVA does not detect statistically significant differences between groups (*p* = 0.325), but bootstrap resampling reveals a significant difference between K and G.

Aluminium (Al): All materials demonstrate progressive accumulation of aluminium ions in adjacent enamel over 12 months. The most substantial increase is observed for Glass Carbomer (+7450%), followed by Fuji IX (+3225%), Equia Forte (+2950%) and Ketac Universal (+1625%). Statistical analyses do not confirm significant intergroup differences (*p* = 0.313).

Silicon (Si): Silicon content increases over time in all groups. The greatest increase occurs in Glass Carbomer at 12 months (+1908.3%), followed by Fuji IX (+375%), Ketac Universal (+266.7%) and Equia Forte (+158.3%). Bootstrap analysis indicates significant differences between Ketac Universal and both Equia Forte and Glass Carbomer (*p* < 0.05).

Strontium (Sr): Strontium enrichment in enamel is evident from one week, particularly in Equia Forte (+290%) and Ketac Universal (+274.1%). By 12 months, Fuji IX exhibits the highest retention (+108.9%). ANOVA and post hoc tests do not show statistically significant differences (*p* = 0.114), though effect size analysis suggests a large effect.

Calcium (Ca) and phosphorus (P): These elements show progressive decreases in enamel concentrations across all groups during the 12-month period.

#### Cumulative Indices of Material–Enamel Interaction

To provide an integrated assessment of material–enamel interaction, four cumulative indices were established based on the peak or 12-month concentrations of selected ions:

- Total Ion Exchange with Enamel (peak concentrations of F, Al, Si, Sr):

This index reflects the maximum ion exchange between the material and enamel, incorporating peak values for fluoride (F), aluminium (Al), silicon (Si) and strontium (Sr).
Ranking: G > F > E > K;

- Long-Term Ion Exchange (12-month concentrations of F, Al, Si, Sr):

This index evaluates ion exchange capacity at the 12-month time point, indicating each material’s ability to sustain ion release over time.
Ranking: G > F > K > E;

- Total Remineralisation Potential (F + Sr only):

Based on the highest measured concentrations of fluoride and strontium, this index highlights the theoretical potential for enamel remineralisation.
Ranking: G > F > K > E;

- Long-Term Remineralisation Activity (12-month concentrations of F + Sr):

This index focuses on the sustained release of fluoride and strontium after 12 months, reflecting long-term bioactivity.
Ranking: G > F > K > E.

### 3.4. EDS Analysis of Chemical Composition Changes in the Dentine Adjacent to the Material

Descriptive and inferential statistical methods were applied to examine temporal trends and inter-material differences in the concentrations (wt.%) of key ions—fluoride (F), aluminium (Al), silicon (Si), phosphorus (P), calcium (Ca) and strontium (Sr)—in dentine adjacent to four glass ionomer cements: Ketac Universal (K), Fuji IX (F), Equia Forte (E) and Glass Carbomer (G). These findings provide valuable insight into ion exchange dynamics at the dentine–material interface, reflecting each material’s biointeractivity and contribution to dentine remineralisation over the 12-month observation period.

**Figure 10:**
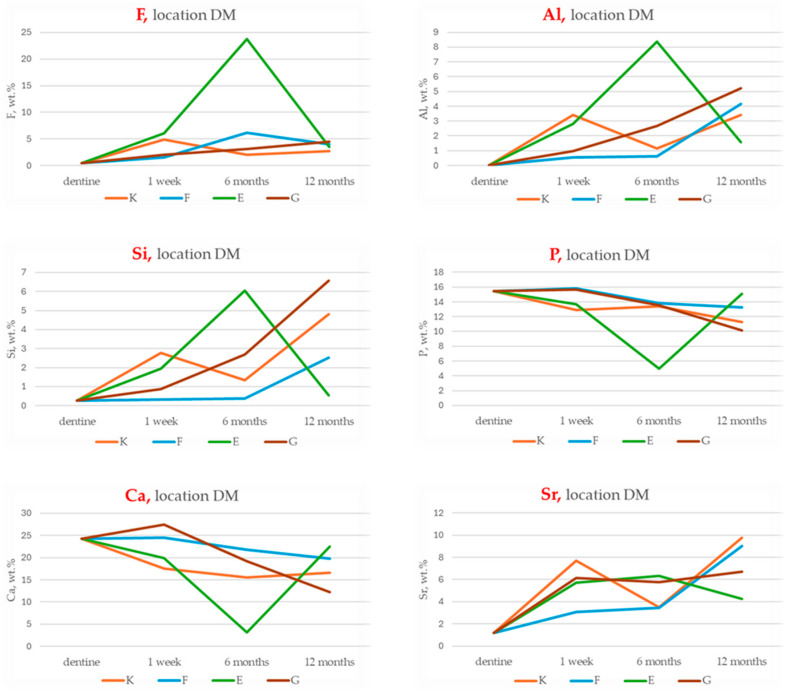
Temporal changes in chemical compositions (wt.%) of fluoride (F), aluminium (Al), silicon (Si), phosphorus (P), calcium (Ca) and strontium (Sr) in dentine adjacent to restorative materials (location DM) of Ketac Universal (K), Fuji IX (F), Equia Forte (E) and Glass Carbomer (G) after 1 week, 6 months and 12 months. Baseline dentine composition (dentine) is included as a reference.

**Figure 11:**
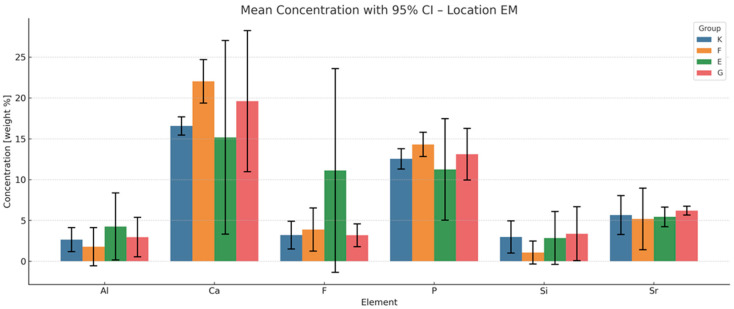
Mean values of chemical compositions (wt.%) of F, Al, Si, P, Ca and Sr in dentine adjacent to the restoration (location DM), averaged across all time points during the 12-month observation period with 95% confidence intervals (CIs) and standard deviations (SDs).

**Figure 12:**
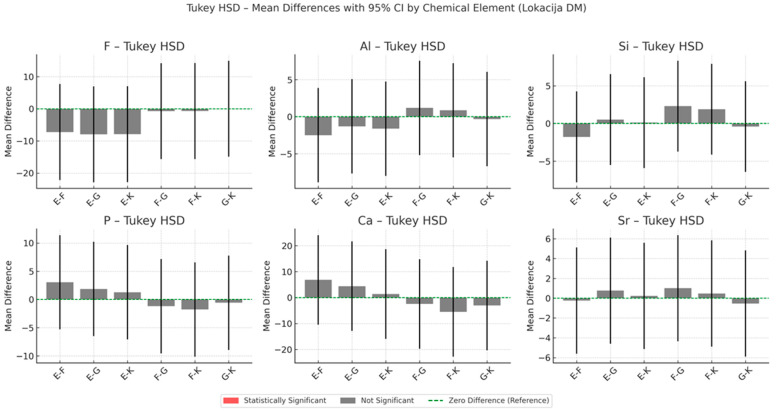
Tukey HSD mean differences with 95% confidence intervals (CIs) for selected chemical elements in the dentine adjacent to the restoration (location DM), averaged across all time points during the 12-month observation period.

The results of energy-dispersive spectroscopy (EDS) performed on dentine adjacent to the restorative materials (location DM) are presented in Figure 10, Figure 11 and Figure 12. Additional detailed data are available in the Supplementary Materials (Tables S5 and S6).

Fluoride (F): All four GICs show increased fluoride concentrations in dentine compared to baseline. The highest enrichment at six months is recorded for Fuji IX (+1157.1%), followed by Glass Carbomer (+526.5%), Ketac Universal (+306.1%) and Equia Forte (+47.9%). Fluoride levels remain elevated at twelve months. Statistically significant differences between materials are observed at one week (*p* = 0.043) and six months (*p* = 0.024).

Aluminium (Al) and silicon (Si): Both elements increase progressively in dentine for all materials. Equia Forte and Fuji IX exhibit the greatest aluminium enrichment, while Glass Carbomer shows the highest relative increase in silicon.

Strontium (Sr): Levels increase consistently over time, with Ketac Universal and Fuji IX showing the highest concentrations at twelve months.

Calcium (Ca) and phosphorus (P): Concentrations of calcium and phosphorus decline across all materials throughout the 12-month period.

#### Cumulative Indices of Material–Dentine Interaction

To summarise the bioactive potential of each material, the following composite indices were calculated based on ion release and remineralisation capacity:

- Total Ion Exchange with Dentine (maximum cumulative concentrations of F, Al, Si, Sr):

E > G > K > F;

- Long-Term Ion Exchange with Dentine (12-month cumulative values of F, Al, Si, Sr):

G > K > F > E;

- Total Remineralisation Potential (Sum of peak F and Sr concentrations):

E > F > K > G;

- Long-Term Remineralisation Activity (12-month F and Sr values):

F > K > G > E.

### 3.5. Comparative Ion Incorporation in Enamel and Dentine

The comparison of maximum ion concentrations between enamel (EM) and dentine (DM) layers revealed consistently higher incorporation of fluoride (F), aluminium (Al), silicon (Si) and strontium (Sr) ions into the dentine across all tested glass ionomer cements—Ketac Universal (K), Fuji IX (F), Equia Forte (E) and Glass Carbomer (G). Statistical analysis using the Kruskal–Wallis H test confirmed significant differences in ion incorporation between enamel and dentine for each ion: fluoride (*p* = 0.005), aluminium (*p* = 0.037), silicon (*p* = 0.034) and strontium (*p* = 0.041). These results are summarised in Table 1, which highlights statistically significant differences (*p* < 0.05). It should be noted that this comparison is based on the highest measured concentrations observed at any time point and does not account for temporal variation.

## 4. Discussion

This study presents a comprehensive evaluation of the year-long biointeractive behaviour of four glass ionomer cements (GICs)—Ketac Universal, Fuji IX, Equia Forte and Glass Carbomer—applied to sound enamel and dentine. Field-emission scanning electron microscopy (FE-SEM) combined with energy-dispersive X-ray spectroscopy (EDS) enabled quantitative assessment of ion exchange (F, Al, Si, Ca, P, Sr) between restorative materials and dental tissues, alongside microstructural characterisation of cement maturation and tooth–material interface evolution. This approach facilitated identification of formulations exhibiting the most sustained therapeutic ion release and tissue incorporation.

FE-SEM analysis reveals a progressive reduction in filler particle density within conventional GICs over time, reflecting partial dissolution or integration into the cement matrix during maturation [27]. By 12 months, the emergence of mesh-like mineralised networks is evident, indicating ongoing mineral deposition consistent with established matrix maturation processes [28]. In contrast, Glass Carbomer distinctly demonstrates discrete, rounded hydroxyapatite (HA) nanoparticles at six months, which subsequently coalesce into well-defined, localised mineralised regions with characteristic radial organisation by 12 months. This observation aligns with findings by Palani et al. [29] and Leung et al. [30], who demonstrated HA-enhanced mineral deposition in modified GICs, and with Zainuddin et al. [31], who confirmed HA-driven nucleation via MAS-NMR. These morphologies, clearly differentiated from preparation artefacts, confirm active biomineralisation and enhanced bioactivity unique to Glass Carbomer [1,9,32]. Minor cracks observed are attributable to mechanical stresses during specimen preparation rather than intrinsic defects.

Consistent with the initial hypothesis, Glass Carbomer exhibits significantly enhanced microstructural evolution, ion release—particularly fluoride and silicon—and superior ion uptake into sound enamel and dentine over the 12-month period compared to conventional GICs.

All tested materials display continuous, pH-responsive release and uptake of fluoride, aluminium, silicon and strontium ions. Statistical analyses reveal significant differences in fluoride incorporation, with Glass Carbomer-treated dentine showing markedly higher fluoride uptake than Fuji IX and Ketac Universal (ANOVA: F(3,8) = 7.62, *p* = 0.012, Cohen’s f = 1.25). Bootstrap confidence intervals reinforce these findings by highlighting broader fluoride content ranges in Glass Carbomer samples [14,33]. Aluminium and silicon levels also vary significantly among materials (*p* < 0.05), whereas calcium and phosphorus concentrations exhibit a progressive decline across all groups over time, suggesting dynamic mineral exchange at the tooth–material interface [34]. Notably, Glass Carbomer maintains elevated fluoride and silicon concentrations in both enamel and dentine after one year, outperforming all conventional formulations.

The temporal increase in fluoride content observed in Equia Forte and Fuji IX aligns with their known capacity for sustained fluoride release, a critical factor for enamel remineralisation and caries prevention [13,35]. Conversely, the initial burst followed by depletion in Glass Carbomer may reflect an advanced ion release mechanism unique to its formulation. The time-dependent decrease in aluminium is likely due to complexation into more stable phases or leaching, influencing matrix cross-linking and maturation [36]. Silicon’s marked increase in Glass Carbomer after six months indicates progressive glass phase dissolution or bioactive silica release, which enhances remineralisation potential [8,10]. Calcium exhibits biphasic kinetics, with an early rise followed by a decline, particularly in Glass Carbomer and Equia Forte, corresponding to mineral deposition and maturation processes [8,37]. Strontium content is consistently higher in Fuji IX, corroborating its role in substituting calcium within apatite lattices to enhance acid resistance and remineralisation—key for patients with high caries risk [38].

These results extend prior shorter-term observations by Murugan et al. [39] and Moheet et al. [8] on ion uptake in nano-hydroxyapatite–silica-modified and conventional GICs. The current data demonstrate that Glass Carbomer not only sustains but in some cases exceeds these incorporation levels over 12 months. Hasan et al. [14] and Lopes et al. [40] similarly noted comparable fluoride release at early stages; however, this study highlights Glass Carbomer’s superior long-term retention of fluoride and strontium in dental tissues.

Ion uptake is consistently greater in dentine than in enamel across all materials (*p* < 0.05), reflecting dentine’s finer hydroxyapatite crystallites, higher organic matrix content and increased permeability. This enhanced reactivity concurs with previous findings by Moheet et al. [8] and Bezerra et al. [41], who attributed such differences to dentine’s tubular microarchitecture and greater surface area facilitating ionic exchange.

Composite indices of cumulative ion exchange and remineralisation potential further underscore Glass Carbomer’s pre-eminence, followed by Fuji IX, with Equia Forte and Ketac Universal displaying comparatively lower performance. This pattern supports the concept that bioactive restorative materials modulate the ionic milieu at both enamel and dentine interfaces, thereby reinforcing dental hard tissues and potentially improving long-term clinical outcomes.

These findings carry important clinical implications. The sustained incorporation of fluoride, strontium and other therapeutic ions into sound enamel and dentine signifies advancing bioactivity in modern GICs. Emerging formulations enhanced with nanofillers, bioactive glass or hydroxyapatite demonstrate improved ion release kinetics and tissue affinity relative to earlier materials [8,9,10,42,43]. This broadens their clinical application beyond carious lesions to include the reinforcement of non-demineralised substrates. Prolonged ion exchange may strengthen intact tissues and contribute to demineralisation prevention, particularly in high caries-risk patients or minimally invasive restorative treatments. Saliva and caries activity, as highlighted by Viana et al. [18], modulate these biological responses, underscoring the necessity for dynamic, clinically relevant testing environments.

While EDS provides valuable compositional insights, its semi-quantitative nature and surface sensitivity impose limitations, including matrix effects, beam penetration depth variability and sample topography influences that may affect interpretation [44,45]. Future studies integrating complementary methods such as Knoop microhardness testing [46], Raman and FTIR spectroscopy [45,47], and confocal microscopy [48] will enable more precise quantification of subsurface mineral changes and strengthen correlations between compositional and mechanical outcomes. This integrative approach promises to deepen understanding of the underlying mechanisms governing GIC bioactivity.

Ultimately, well-designed in vivo investigations and advanced specimen preparation techniques are essential to elucidate ion transport mechanisms and assess restoration integrity under physiological conditions. Addressing these challenges will advance the development of next-generation bioactive restorative materials, fulfilling the overarching goal of enhancing restorative outcomes through optimised material–tissue interactions.

## 5. Conclusions

In conclusion, this study demonstrates that Glass Carbomer exhibits markedly superior bioactivity compared to conventional glass ionomer cements, forming hydroxyapatite nanoparticles by six months and extensive mineralised regions by twelve months, while achieving the highest fluoride and silicon uptake in both enamel and dentine. Among the conventional GICs, Equia Forte released the greatest intra-matrix fluoride, Fuji IX delivered the most strontium and Ketac Universal exhibited the lowest sustained ion release. Dentine consistently showed greater ion incorporation than enamel, likely due to its higher permeability and organic content. These findings confirm that advanced GIC formulations can reinforce sound dental tissues through prolonged ion exchange and biomineralisation. Future research should incorporate complementary analytical techniques (e.g., microhardness testing, Raman and FTIR spectroscopy) and in vivo investigations under dynamic oral conditions to validate and extend these in vitro findings.

## Figures and Tables

**Table 1:** *p*-values (Kruskal–Wallis H test) and mean differences in maximum concentrations (wt.%) of fluoride (F), aluminium (Al), silicon (Si) and strontium (Sr) between dentine (DM) and enamel (EM) regions adjacent to restorative materials. Statistically significant differences (*p* < 0.05) are highlighted by grey area.

DM vs. EM	F		Al		Si		Sr	
	*p* Value	Mean Differences	*p* Value	Mean Differences	*p* Value	Mean Differences	*p* Value	Mean Differences
K	0.001	+1.98	0.027	+2.47	0.059	+1.45	0.046	+2.00
F	0.027	+2.43	0.508	+0.07	1.000	+0.51	0.137	+0.22
E	0.003	+5.30	0.010	+2.62	0.020	+2.23	0.002	+3.12
G	0.600	+0.37	0.183	−0.05	0.656	+0.49	0.008	+2.37

## Data Availability

The original contributions presented in this study are included in the article/supplementary material. Further inquiries can be directed to the corresponding author.

## Supplementary Materials

The following supporting information can be downloaded at: https://www.mdpi.com/article/10.3390/ma18153580/s1, Table S1: Chemical compositions (wt.%) of fluoride (F), aluminium (Al), silicon (Si), phosphorus (P), calcium (Ca) and strontium (Sr) in Ketac Universal (K), Fuji IX (F), Equia Forte (E) and Glass Carbomer (G) after 1 week (1), 6 months (6) and 12 months (12) of maturation; Table S2: Mean values of chemical compositions (wt.%), standard deviations (SDs) and 95% confidence intervals (CIs) for each material in the material region (location M), averaged across all time points during the 12-month observation period; Table S3: Mean concentrations (wt.%) and standard deviations (SDs) of selected ions—fluoride (F), aluminium (Al), silicon (Si), phosphorus (P), calcium (Ca) and strontium (Sr)—in enamel adjacent to the material (location EM) for Ketac Universal (K), Fuji IX (F), Equia Forte (E) and Glass Carbomer (G) after 1 week (1), 6 months (6) and 12 months (12). Baseline ion concentrations in sound enamel (E) are included for reference; Table S4: Mean values of chemical compositions (wt.%), standard deviations (SDs) and 95% confidence intervals (CIs) for each material in enamel adjacent to the restoration (location EM), averaged across all time points during the 12-month observation period; Table S5: Mean concentrations (wt.%) and standard deviations (SDs) of selected ions (F, Al, Si, P, Ca, Sr) in the dentine adjacent to the material (location DM) for Ketac Universal (K), Fuji IX (F), Equia Forte (E) and Glass Carbomer (G) after 1 week (1), 6 months (6) and 12 months (12). Baseline ion concentrations in sound dentine (D) are included for comparison; Table S6: Mean values of chemical compositions (wt.%), standard deviations (SDs) and 95% confidence intervals (CIs) for each material in dentine adjacent to the restoration (location DM), averaged across all time points during the 12-month observation period.

## Abbreviations

The following abbreviations are used in this manuscript:

ANOVA	Analysis of Variance
ART	Atraumatic Restorative Treatment
CI	Confidence Interval
CT	Computed Tomography
DM	Dentine adjacent to Material
E	Equia Forte
E1, E6, E12	Equia Forte at 1 week, 6 months and 12 months respectively
EDS	Energy Dispersive X-ray Spectroscopy
EM	Enamel adjacent to Material
F	Fuji IX
F1, F6, F12	Fuji IX at 1 week, 6 months and 12 months, respectively
FE-SEM	Field-Emission Scanning Electron Microscopy
G	Glass Carbomer
G1, G6, G12	Glass Carbomer at 1 week, 6 months and 12 months, respectively
GIC(s)	Glass Ionomer Cement(s)
HA	Hydroxyapatite
K	Ketac Universal
K1, K6, K12	Ketac Universal at 1 week, 6 months and 12 months, respectively
MR	Mineralised Region
NaOCl	Sodium Hypochlorite
SD	Standard Deviation
SEM	Scanning Electron Microscopy
wt.%	Weight Percent
