# Supplementary material for: Bioactivity of Glass Carbomer Versus Conventional GICs in Sound Enamel and Dentine: A 12-Month SEM-EDS Study
Source: Materials (Basel). 2025 Jul 30;18(15):3580. doi: 10.3390/ma18153580 (PMC12348236; doi:10.3390/ma18153580)
Supplement: Supplementary file 1 [file materials-18-03580-s001.zip › materials-3731113-supplementary.pdf]

Supplementary Material

Table S1. Chemical compositions (wt.%) of fluoride (F), aluminium (Al), silicon (Si), phosphorus (P), calcium (Ca) and strontium (Sr) in Ketac Universal (K), Fuji IX (F), Equia Forte (E) and Glass Carbomer (G) after 1 week (1), 6 months (6) and 12 months (12) of maturation.

**M**	**F**	**Al**	**Si**	**P**	**Ca**	**Sr**	**Rest**
				**wt.%**			
K1	10.12	9.97	14.07	1.85	0.60	15.83	47.56
K6	10.58	10.45	14.17	2.20	1.18	15.76	45.66
K12	12.42	9.67	12.16	2.01	0.69	15.21	47.84
F1	11.10	12.44	12.70	2.33	1.36	19.10	40.97
F6	14.78	11.26	11.02	2.88	2.46	16.81	40.79
F12	12.04	11.44	11.33	2.40	0.99	19.27	43.53
E1	14.74	12.44	12.91	2.46	1.28	11.80	44.37
E6	19.19	11.79	11.25	2.22	1.50	10.43	40.62
E12	21.08	10.96	9.62	2.05	0.68	10.24	45.37
G1	13.24	9.12	9.02	3.48	2.89	16.08	46.17
G6	8.36	9.88	16.02	2.55	3.58	8.88	50.73
G12	12.25	8.53	12.33	2.35	2.18	7.98	54.38

Table S2. Mean values of chemical compositions (wt.%), standard deviations (SDs) and 95% confidence intervals (CIs) for each material in the material region (location M), averaged across all time points during the 12-month observation period.

M	Element	Mean (wt.%) ± SD	95% CI Lower	95% CI Upper
K	F	11.04 ± 1.22	10.12	12.42
	Al	10.03 ± 0.39	9.67	10.45
	Si	13.47 ± 1.13	12.16	14.17
	P	2.02 ± 0.18	1.85	2.20
	Ca	0.82 ± 0.31	0.60	1.18
	Sr	15.60 ± 0.34	15.21	15.83
F	F	12.64 ± 1.91	11.10	12.78
	Al	11.71 ± 0.64	11.26	12.84
	Si	11.68 ± 0.89	11.02	12.70
	P	2.54 ± 0.30	2.33	2.88
	Ca	1.60 ± 0.76	0.99	2.46
	Sr	18.39 ± 1.37	16.81	19.27
E	F	18.34 ± 3.26	14.78	21.90
	Al	11.73 ± 0.74	10.95	12.51
	Si	11.26 ± 1.65	9.62	12.90
	P	2.24 ± 0.21	2.05	2.42
	Ca	1.15 ± 0.42	0.68	1.62
	Sr	10.82 ± 0.85	10.24	11.80
G	F	11.28 ± 2.58	8.36	14.20
	Al	9.18 ± 0.68	8.53	9.83
	Si	12.46 ± 3.50	9.02	15.90
	P	2.79 ± 0.60	2.35	3.48
	Ca	2.88 ± 0.70	2.18	3.58
	Sr	10.98 ± 4.44	7.98	16.08

Table S3. Mean concentrations (wt.%) and standard deviations (SDs) of selected ions—fluoride (F), aluminium (Al), silicon (Si), phosphorus (P), calcium (Ca) and strontium (Sr)—in enamel adjacent to the material (location EM) for Ketac Universal (K), Fuji IX (F), Equia Forte (E) and Glass Carbomer (G) after 1 week (1), 6 months (6) and 12 months (12). Baseline ion concentrations in sound enamel (E) are included for reference.

EM		F	Al	Si	P	Ca	Sr	Rest
					**Mean (wt.%) ± SD**			
	E	0.18 ± 0.21	0.04 ± 0.08	0.24 ± 0.02	17.95 ± 0.29	33.27 ± 0.87	1.12 ± 0.20	47.29 ± 0.95
	K1	0.91 ± 0.13	0.59 ± 0.14	0.85 ± 0.25	17.28 ± 0.82	31.27 ± 1.28	2.77 ± 0.44	46.33 ± 1.74
	K6	1.27 ± 0.06	0.53 ± 0.05	0.88 ± 0.13	16.15 ± 0.62	26.31 ± 0.44	2.11 ± 0.40	52.77 ± 1.30
	K12	0.77 ± 0.28	0.69 ± 0.27	0.87 ± 0.58	15.24 ± 0.95	30.86 ± 7.78	4.19 ± 1.25	47.37 ± 6.97
	F1	0.50 ± 0.09	0.22 ± 0.21	0.50 ± 0.37	16.97 ± 0.49	29.24 ± 3.94	2.12 ± 0.39	50.46 ± 4.03
	F6	3.58 ± 0.32	1.03 ± 0.55	1.14 ± 0.57	16.54 ± 0.54	29.12 ± 0.19	3.22 ± 0.94	45.37 ± 1.01
	F12	1.99 ± 1.06	1.33 ± 0.43	0.67 ± 0.17	16.97 ± 0.93	28.77 ± 6.95	3.69 ± 1.11	46.59 ± 5.27
	E1	0.57 ± 0.23	0.31 ± 0.11	0.29 ± 0.02	18.17 ± 0.75	33.75 ± 0.89	1.36 ± 0.04	45.54 ± 1.59
	E6	2.87 ± 0.68	0.74 ± 0.09	0.54 ± 0.11	17.11 ± 0.18	29.13 ± 0.41	1.98 ± 0.10	47.64 ± 0.97
	E12	2.11 ± 0.37	1.22 ± 0.36	0.62 ± 0.27	16.58 ± 0.54	30.67 ± 0.97	2.34 ± 0.23	46.45 ± 0.31
	G1	2.43 ± 2.42	1.37 ± 1.24	1.32 ± 1.10	13.91 ± 4.26	21.84 ± 9.17	4.37 ± 2.95	54.77 ± 6.99
	G6	2.93 ± 2.43	0.59 ± 0.40	1.37 ± 0.90	17.71 ± 1.03	36.38 ± 5.86	2.34 ± 0.48	39.65 ± 5.92
	G12	2.39 ± 0.84	3.02 ± 1.30	4.82 ± 1.79	13.00 ± 1.47	23.17 ± 2.99	3.60 ± 1.31	49.99 ± 1.18

Table S4. Mean values of chemical compositions (wt.%), standard deviations (SDs) and 95% confidence intervals (CIs) for each material in enamel adjacent to the restoration (location EM), averaged across all time points during the 12-month observation period.

EM	Element	Mean (wt.%) ± SD	95% CI Lower	95% CI Upper
K	F	0.98 ± 0.26	0.34	1.62
	Al	0.60 ± 0.08	0.40	0.80
	Si	0.87 ± 0.02	0.83	0.90
	P	16.22 ± 1.02	13.68	8.76
	Ca	29.48 ± 2.75	22.64	36.32
	Sr	2.12 ± 0.64	0.53	3.71
F	F	2.02 ± 1.54	-1.80	5.85
	Al	0.86 ± 0.57	-0.57	2.29
	Si	0.77 ± 0.33	-0.05	1.59
	P	16.83 ± 0.25	16.21	17.44
	Ca	29.04 ± 0.24	28.44	29.65
	Sr	3.01 ± 0.80	1.03	5.00
E	F	1.85 ± 1.17	-1.06	4.76
	Al	0.69 ± 0.36	-0.20	1.58
	Si	0.51 ± 0.21	-0.01	1.04
	P	16.99 ± 1.27	13.83	20.16
	Ca	31.14 ± 2.37	25.26	37.02
	Sr	2.21 ± 0.80	0.23	4.19
G	F	2.58 ± 0.30	1.84	3.33
	Al	1.66 ± 1.24	-1.42	4.74
	Si	2.38 ± 2.12	-2.88	7.64
	P	15.64 ± 2.41	9.66	21.62
	Ca	27.13 ± 8.04	7.16	47.10
	Sr	3.44 ± 1.03	0.89	5.99

Table S5. Mean concentrations (wt.%) and standard deviations (SDs) of selected ions (F, Al, Si, P, Ca, Sr) in the dentine adjacent to the material (location DM) for Ketac Universal (K), Fuji IX (F), Equia Forte (E) and Glass Carbomer (G) after 1 week (1), 6 months (6) and 12 months (12). Baseline ion concentrations in sound dentine (D) are included for comparison.

DM	F	Al	Si	P	Ca	Sr	Rest
				Mean (wt.%) ± SD			
D	0.49 ± 0.19	0.00 ± 0.00	0.26 ± 0.08	15.43 ± 0.46	24.25 ± 1.98	1.20 ± 0.18	59.39 ± 2.23
K1	4.88 ± 0.83	3.40 ± 2.01	2.77 ± 2.01	12.91 ± 1.05	17.52 ± 0.94	7.70 ± 3.35	50.83 ± 6.21
K6	1.99 ± 0.26	1.14 ± 1.15	1.33 ± 1.37	13.41 ± 0.68	15.54 ± 2.64	3.49 ± 2.26	63.10 ± 4.44
K12	2.75 ± 1.16	3.40 ± 0.72	4.80 ± 2.41	11.30 ± 2.73	16.63 ± 5.09	9.79 ± 2.12	51.31 ± 4.07
F1	1.49 ± 0.28	0.5 4± 0.14	0.33 ± 0.03	15.78 ± 0.83	24.48 ± 3.19	3.07 ± 0.94	54.30 ± 4.23
F6	6.16 ± 1.41	0.62 ± 0.49	0.39 ± 0.49	13.86 ± 1.99	21.82 ± 3.49	3.47 ± 1.72	53.67 ± 2.81
F12	4.01 ± 0.91	4.16 ± 0.79	2.51 ± 1.36	13.28 ± 1.00	19.80 ± 3.09	9.02 ± 0.20	47.22 ± 2.31
E1	6.10 ±1.54	2.81 ± 0.66	1.94 ± 0.58	13.70 ± 1.05	19.90 ± 1.12	5.73 ± 0.91	49.84 ± 1.52
E6	23.75 ± 1.49	8.38 ± 1.07	6.05 ± 0.63	4.96 ± 1.33	3.16 ± 1.41	6.31 ± 0.04	47.41 ± 0.41
E12	3.48 ± 0.62	1.58 ± 1.16	0.56 ± 0.35	15.10 ± 0.21	22.46 ± 2.66	4.26 ± 1.41	52.57 ± 6.41
G1	2.00 ± 0.22	0.98 ± 0.15	0.86 ± 0.24	15.66 ± 0.66	27.45 ± 1.21	6.14 ± 0.28	46.91 ± 1.37
G6	3.07 ± 0.65	2.67 ± 0.88	2.68 ± 1.16	13.56 ± 0.65	19.15 ± 0.86	5.75 ± 0.65	53.11 ± 3.37
G12	4.47 ± 0.59	5.23 ± 1.53	6.57 ± 1.60	10.12 ± 1.36	12.21 ± 3.28	6.70 ± 0.64	54.71 ± 0.30

Table S6. Mean values of chemical compositions (wt.%), standard deviations (SDs) and 95% confidence intervals (CIs) for each material in dentine adjacent to the restoration (location DM), averaged across all time points during the 12-month observation period.

DM	Element	Mean (wt.%) ± SD	95% CI Lower	95% CI Upper
K	F	3.21 ± 1.50	-0.51	6.93
	Al	2.65 ± 1.30	-0.59	5.89
	Si	2.97 ± 1.74	-1.36	7.30
	P	12.54 ± 1.10	9.80	15.28
	Ca	16.56 ± 0.99	14.10	19.03
	Sr	5.66 ± 2.11	0.42	10.90
F	F	3.89 ± 2.34	-1.92	9.69
	Al	1.77 ± 2.07	-3.36	6.91
	Si	1.08 ± 1.24	-2.01	4.16
	P	14.31 ± 1.31	11.06	7.56
	Ca	22.03 ± 2.35	16.20	27.86
	Sr	5.19 ± 3.33	-3.08	13.45
E	F	11.11 ± 11.02	-16.28	38.50
	Al	4.26 ± 3.62	-4.74	13.26
	Si	2.85 ± 2.86	-4.24	9.94
	P	11.25 ± 5.49	-2.40	24.90
	Ca	15.17 ± 10.48	-10.87	41.21
	Sr	5.43 ± 1.06	2.81	8.06
G	F	3.18 ± 1.24	0.10	6.26
	Al	2.96 ± 2.14	-2.36	8.28
	Si	3.37 ± 2.92	3.88	10.62
	P	13.11 ± 2.80	6.17	20.06
	Ca	19.60 ± 7.63	0.65	38.56
	Sr	6.20 ± 0.48	5.01	7.38
